# Constitutive internalisation of EP2 differentially regulates G protein signalling

**DOI:** 10.1530/JME-23-0153

**Published:** 2024-05-15

**Authors:** Abigail R Walker, Holly A Parkin, Sung Hye Kim, Vasso Terzidou, David F Woodward, Phillip R Bennett, Aylin C Hanyaloglu

**Affiliations:** 1 Institute of Reproductive and Developmental Biology, Department Metabolism, Digestion and Reproduction, Imperial College London, London, UK; 2 Department of Bioengineering, Imperial College London, London, UK

**Keywords:** EP2, GPCR, prostaglandin, trafficking

## Abstract

The prostanoid G protein-coupled receptor (GPCR) EP2 is widely expressed and implicated in endometriosis, osteoporosis, obesity, pre-term labour and cancer. Internalisation and intracellular trafficking are critical for shaping GPCR activity, yet little is known regarding the spatial programming of EP2 signalling and whether this can be exploited pharmacologically. Using three EP2-selective ligands that favour activation of different EP2 pathways, we show that EP2 undergoes limited agonist-driven internalisation but is constitutively internalised via dynamin-dependent, β-arrestin-independent pathways. EP2 was constitutively trafficked to early and very early endosomes (VEE), which was not altered by ligand activation. APPL1, a key adaptor and regulatory protein of the VEE, did not impact EP2 agonist-mediated cAMP. Internalisation was required for ~70% of the acute butaprost- and AH13205-mediated cAMP signalling, yet PGN9856i, a Gαs-biased agonist, was less dependent on receptor internalisation for its cAMP signalling, particularly in human term pregnant myometrial cells that endogenously express EP2. Inhibition of EP2 internalisation partially reduced calcium signalling activated by butaprost or AH13205 and had no effect on PGE2 secretion. This indicates an agonist-dependent differential spatial requirement for Gαs and Gαq/11 signalling and a role for plasma membrane-initiated Gαq/11-Ca^2+^-mediated PGE2 secretion. These findings reveal a key role for EP2 constitutive internalisation in its signalling and potential spatial bias in mediating its downstream functions. This, in turn, could highlight important considerations for future selective targeting of EP2 signalling pathways.

## Introduction

EP2 is a prostaglandin E2 (PGE2) receptor and one of four G protein-coupled receptors (GPCRs) activated by PGE2 (EP1-4). EP2 is widely expressed, with high expression in vascular endothelium, human intestine, lung, kidney, uterus, and cerebral cortex (Woodward *et al.* 2011), and is implicated in numerous pathological disorders via both under and over-activation, including epilepsy, endometriosis and cancer. Despite this, EP2 is not currently exploited therapeutically, which may reflect a need to selectively target EP2 activity.

GPCR function is tightly integrated with receptor subcellular location (Calebiro *et al.* 2009, Tsvetanova *et al.* 2017, Gorvin *et al.* 2018, Tsvetanova *et al.* 2021). Thus, GPCR internalisation is no longer accepted as synonymous with signal termination. In contrast, receptors can signal not only from the plasma membrane but from distinct subcellular compartments, including early endosomes (Jean-Alphonse *et al.* 2014, Irannejad *et al.* 2017). Following ligand-induced internalisation, we have demonstrated that GPCRs are sorted into distinct endosomal compartments, termed very early endosomes (VEE), that not only dictate their post-endocytic sorting fate but also impact receptor activity (Sposini *et al.* 2017, Caengprasath *et al.* 2020).

Whilst EP2 is classically a Gαs-coupled receptor, in distinct cell types EP2 can also activate Gαq/11–Ca^2+^ pathways (Kandola *et al.* 2014, Kishore *et al.* 2017, Walker *et al.* 2022). Interestingly, ligand-selective agonists of EP2 (butaprost, AH13205 and PGN9856/i) may exhibit bias in primary myometrial cells and HEK 293 cells, either activating both Gαs and Gαq/11 pathways (butaprost, AH13205) or only Gαs (PGN9856i) (Walker *et al.* 2022). For the latter agonist, PGN9856 and its isopropyl ester, are not only highly biased towards Gαs but also exhibit long-lasting actions in the context of glaucoma (Woodward *et al.* 2019). The mechanism behind the multiplicity of butaprost, AH13205 and PGN9856 signalling is unknown, but it is possible that there is spatial bias at the root of the observed differences, as has been seen for other GPCRs (Jean-Alphonse *et al.* 2018).

Previous studies on EP2 trafficking have primarily addressed the activation of EP2 with its endogenous ligand, PGE2 (Desai *et al.* 2000, Penn *et al.* 2001, Nunez *et al.* 2020). These studies have shown that EP2 undergoes limited agonist-induced internalisation and, due to its short C-terminus, does not interact with β-arrestins, a family of adaptor proteins that are classically associated with GPCR internalisation, signal termination and signal activation. Whether distinct ligands for EP2, including non-prostanoid agonists with different structures, may induce different trafficking profiles and the role of such trafficking on G protein signalling, is unknown. Thus, in this study, we have profiled EP2 internalisation and endosomal trafficking in HEK 293 cells using three highly selective EP2 ligands that have distinct signalling profiles.

## Materials and methods

### Cell lines

HEK 293 cells (ATCC) and β-arrestin 1/2 knockout cells (kindly gifted by Asuka Inoue) were maintained in DMEM with phenol red, supplemented with 10% fetal bovine serum and 100 U/mL penicillin–streptomycin. Cells were cultured in 75 cm flasks at 37°C in 5% CO_2_. Cells were passaged upon reaching 80–90% confluency, using 0.25% trypsin with 0.02% EDTA in phosphate-buffered saline. Cells were transfected using lipofectamine 2000 (Invitrogen) in DMEM with phenol red, supplemented with 10% fetal bovine serum, for 72 h before experiments.

### Primary myometrial cells

Myometrial tissue was acquired at Chelsea and Westminster Hospital or Queen Charlotte’s and Chelsea Hospital, London, UK. Tissues were obtained from term (38 + 0–40 weeks gestation) pregnant women undergoing elective caesarean from the upper margin of the incision made at the lower segment of the uterus, before the onset of labour. Tissue was only taken from uncomplicated, singleton pregnancies.

The studies involving human participants were reviewed and approved by the Riverside Ethics Committee (Ref. 3358) and the London Harrow Research Ethics Committee (Ref. 19/LO/1657). The patients/participants provided their written informed consent to participate in this study.

### Reagents and plasmids

The human EP2 cDNA was amplified from the HA-tagged human EP2 construct (in pcDNA 3.1), kindly provided by Barrie Ashby (Temple University School of Medicine). This was ligated into pcDNA3.1 following digestion of both vectors and inserted with AFEI and XBAI to yield the FLAG-EP2 plasmid. FLAG-B2AR was gifted by Mark Von Zastrow (University of California, San Francisco).

Antibodies used were mouse anti-β-arrestin 1/2 (Thermo Fisher Scientific); mouse anti-GAPDH (Millipore); goat anti-mouse horseradish peroxidase (HRP) (Thermo Fisher Scientific); anti-FLAG M1 antibody (Sigma); anti-EEA1 (Cell Signaling); anti-APPL1 (Cell Signaling); Alexa Fluor goat anti-rabbit 488 (Invitrogen) and Alexa Fluor goat anti-mouse 555 (Invitrogen).

Inhibitors used were dyngo-4a (Abcam), used at 50 μM, and 3-isobutyl-1-methylxanthine (IBMX) (Sigma), used at 0.5 mM (5 min pretreatment). Butaprost, AH-13205 and isoproterenol were from Sigma-Aldrich and were used at 10 μM. Oxytocin was from Sigma and used at 100 nM. PGN9856i (PGN9856 isopropyl ester) and PGN9856 (PGN9856 free acid) were an unrestricted gift from Allergan Inc. and were used at 100 nM.

### Measurement of intracellular Ca^2+^

Cells were seeded into 35 mm round dishes with 14 mm × 1.5 mm glass coverslips. Cells were incubated with Fluo-4 AM Ca^2+^ indicator (Thermo Fisher) for 30 min at 37˚C and 30 min at room temperature, as per the manufacturer’s instructions. For the 45 min dyngo-4a pretreatment, cells were incubated with dyngo-4a for 15 min prior to the 30 min incubation with Fluo-4AM Ca^2+^ indicator with dyngo-4a. Time-series images were acquired using a Leica SP5 confocal microscope, Leica LAS AF image acquisition software and a 488 nm excitation laser. The basal signal was measured before the addition of ligand. Raw data were analysed using the Fiji Time series analyzer plugin. The maximal intensity was measured in duplicate for at least 30 cells per condition, and the basal intensity of each cell was subtracted. This was then averaged across cells in each condition.

### cAMP accumulation assay

Cells were pre-treated with 0.5 mM IBMX for 5 min before the addition of ligand, and IBMX for a further 5 min. Cells were lysed and assayed as per the manufacturer’s instructions (Cisbio, Codolet, Occitania, France).

### Confocal microscopy

After reaching 60–70% confluency on glass coverslips, cells were incubated with anti-FLAG antibody for 20 min before ligand stimulation. Cells were then washed three times with PBS Ca^2+^. For imaging of endosomal markers, the plasma membrane signal was stripped by four washes with PBS + 0.04 M EDTA. Cells were immediately fixed with 4% PFA for 20 min and washed three times for 5 min with PBS Ca^2+^. Cells were permeabilised for 20 min in PBS Ca^2+^ + 0.2% Triton, and washed three times with PBS Ca^2+^ before blocking for 30 min in PBS Ca^2+^ + 2% FBS. If required, cells were then incubated for 1 h at RT with primary antibodies for endosomal markers (APPL1 and EEA1) before three PBS Ca^2+^ washes. Cells were then incubated for 1 h at RT with secondary antibodies before washing with PBS Ca^2+^ mounted onto slides using Fluoromount-G and sealed with clear nail varnish. Samples were kept in the dark at 4°C until imaging using a Leica SP5 confocal microscope with a 63 1.4 numerical aperture (NA) objective. Raw image files were analysed using ImageJ.

### Western blot

Cells at 70–95% confluency were lysed in 100 μL lysis buffer per 6-well plate containing: 50 mM Tris–HCl (pH 7.5), 0.5 mM EDTA, 150 mM NaCl, 1% (v/v) Triton X-100, 1 mM PMSF, 1 mM NaF, 1 mM NaVO_3_ and one cOmplete EDTA-free protease inhibitor tablet. 20–50 μg lysate, as determined using Bradford assay, plus Laemmli buffer (0.5M pH 6.8 Tris, 10% SDS, 1% bromophenol blue, 2% β-mercaptoethanol, 20% glycerol) were separated with 1× SDS running buffer at 140 V on SDS-PAGE gels polymerised with TEMED and ammonium persulfate: separating gel (12% polyacrylamide, 380 mM pH 8.8 Tris, 0.1% SDS), resolving gel (5% poly, 130 mM Tris (pH 6.8), 0.1% SDS). Following transfer onto nitrocellulose membranes, membranes were blocked in 5% milk TBS-T for 30 min and incubated overnight at 4°C with the primary antibody in blocking buffer. Membranes were washed with TBS-T and incubated in TBS-T with appropriate secondary antibodies for 1 h before signal detection using HRP substrate and a chemiluminescent imager (ImageQuant LAS 4000).

### Measurement of secreted PGE2

HEK 293 cells stably expressing EP2 were cultured in 12-well plates until 80–90% confluent. They were then treated with ligand in 1 mL media for either 5 min or 1 h. Media was collected, and PGE2 concentrations ascertained using ELISA (Enzo Lifesciences, Farmingdale, NY, USA), as per the manufacturer's protocol.

### Flow cytometry

Cells were cultured in 12-well plates until 70–80% confluent. For assessment of ligand-induced internalisation, cells were incubated with anti-FLAG antibody for 20 min and treated with a ligand at 37°C before staining with a secondary antibody on ice for 1 h. For measurements of constitutive internalisation, cells were incubated with FLAG antibody for 1 h at 4°C before one condition was brought to 37°C for 1 h to allow resumption of internalisation. All conditions were then incubated with a secondary antibody at 4°C before membrane fluorescence was quantified. Plasma membrane fluorescence was measured using the FACSCalibur Flow Cytometer (BD Biosciences).

### Statistical analysis

All statistical data analyses were performed in GraphPad Prism 8. For the comparison of two groups, two-tailed Student’s *t*-test was performed to determine statistical significance. In cases where data were analysed as fold change over basal or agonist response, a one-sample *t*-test was performed to allow comparison against a bounded value. For multiple comparisons, one-way ANOVA was used with either Dunnett’s, Sidak’s or Tukey’s *post* *hoc* analysis for comparison either against a single control group, all groups against one another or preselected groups. For each test, *P* < 0.05 is considered significant. Data are shown as mean ± s.e.m. from at least three independent experiments unless indicated in the figure legend.

## Results

### EP2 undergoes constitutive but limited agonist-driven internalisation

**Figure 1:**
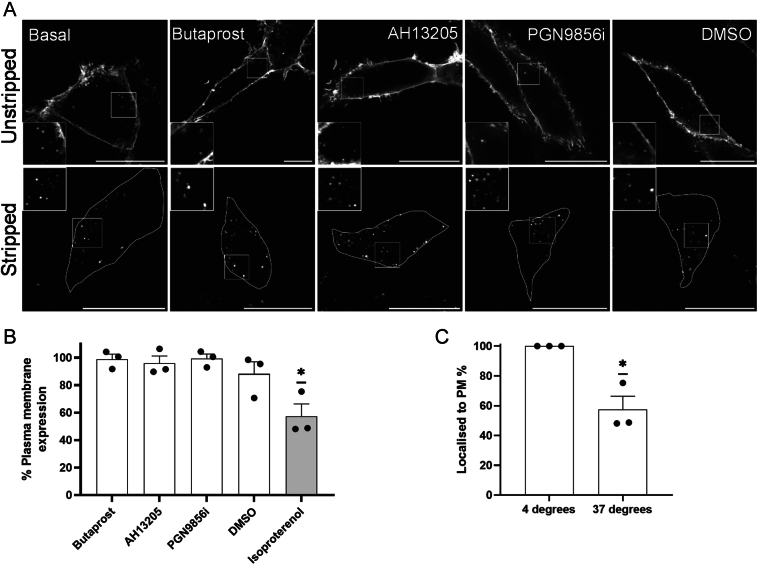
EP2 undergoes constitutive but not agonist-driven internalisation. (A) HEK 293 cells expressing FLAG-EP2 live-labelled with anti-FLAG Ab and treated with butaprost (10 μM), AH13205 (10 μM), PGN9856i (100 nM) or DMSO for 20 min ± EDTA stripping of plasma membrane signal. Cells were fixed, permeabilised and labelled with immunofluorescent secondary antibodies. Cells were imaged by confocal microscopy, and representative images shown from at least three independent experiments. Scale bar: 20 μm, inset size: 7 μm. (B) HEK 293 cells expressing FLAG-EP2 were live-labelled with anti-FLAG Ab and stimulated for 20 min with butaprost (10 μM), AH13205 (10 μM), PGN9856i (100 nM) or DMSO, and cell surface expression measured via flow cytometry. Data are shown as percentage change from basal surface expression, represented as mean ± s.e.m., *n* = 3. One-sample *t*-test: *P < 0.05. (C) HEK 293 cells expressing FLAG-EP2 live-labelled with anti-FLAG M1 antibody at 4°C with/without 1 h incubation at 37°C. Cell surface expression measured via flow cytometry. Data are shown as percentage change from surface expression in cells kept at 4°C, represented as mean ± s.e.m. *n* = 3. One-sample *t*-test: *P < 0.05.

EP2 agonists have significant signalling bias, towards either Gαs-mediated cAMP pathways or pro-inflammatory Gαq/11 pathways (Walker *et al.* 2022). Whilst it has previously been shown that EP2 does not undergo internalisation following activation with its endogenous ligand, PGE2 (Desai *et al.* 2000), whether EP2 ligands which are known to induce functionally different receptor signals exhibit distinct receptor trafficking profiles is currently unknown. The ability of distinct EP2 ligands to induce receptor internalisation was first assessed. HEK 293 cells stably expressing FLAG-tagged human EP2 were treated with either butaprost, AH13205, PGN9856i or DMSO, and receptors were assessed both qualitatively via confocal microscopy (Fig. 1A) and quantitatively via flow cytometry (Fig. 1B). Agonists were used at concentrations yielding the maximal cAMP response (Supplementary Fig. 1, see section on supplementary materials given at the end of this article) and reported previously for butaprost and PGN9856i (Walker *et al.* 2022). Confocal imaging revealed that EP2 did not exhibit enhanced internalisation following treatment with any of the ligands. This was confirmed by flow cytometry and compared to the β2-adrenergic receptor (β2AR), known to undergo rapid and robust ligand-induced internalisation (Moore *et al.* 1995, Seachrist *et al.* 2000, Jean-Alphonse *et al.* 2014). Whilst isoproterenol induced significant internalisation of the β2AR as measured by flow cytometry, there was no change in EP2 internalisation between the untreated and ligand-treated conditions, consistent with prior reports that EP2 does not undergo ligand-mediated internalisation (Desai *et al.* 2000, Penn *et al.* 2001). However, as receptor-containing endosomes could be observed under basal conditions (Fig. 1A), constitutive internalisation was quantified using flow cytometry. Labelling of FLAG-EP2 at the plasma membrane at 4°C followed by resumption of internalisation at 37°C demonstrated that ~40% of EP2 receptors were found to internalise constitutively (Fig. 1C).

#### Constitutive EP2 internalisation is β-arrestin independent but dynamin dependent

**Figure 2:**
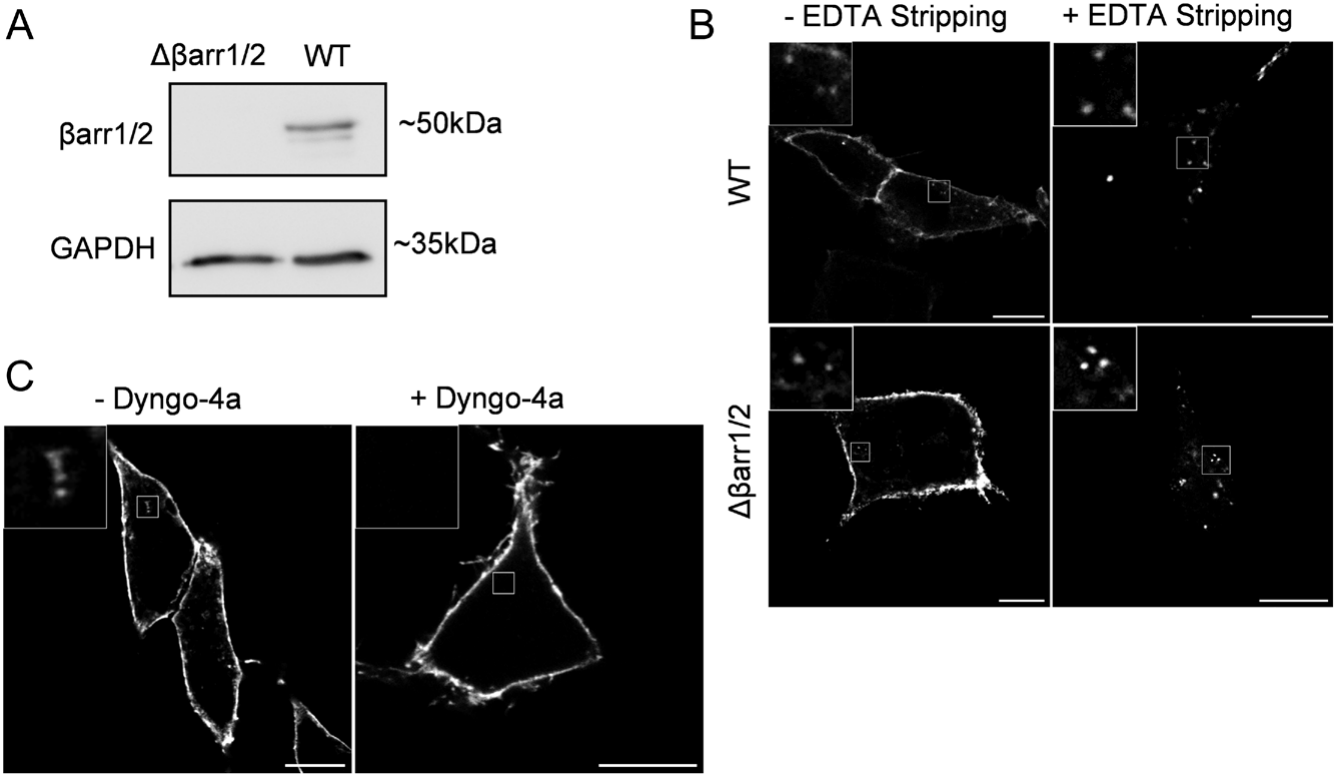
Internalisation of EP2 occurs via β-arrestin independent, dynamin-dependent pathways. (A) WT HEK 293 cells and cells lacking β-arrestin 1/2 were checked via western blot for β-arrestin 1/2 expression (~50 kDa) using GAPDH as a loading control (~35 kDa). Representative image is shown. (B) Confocal microscopy images of FLAG-EP2 expressing WT HEK 293 cells and cells lacking β-arrestin 1/2. Cells were labelled live with anti-FLAG M1 antibody ± EDTA stripping of plasma membrane signal before fixation and stained with immunofluorescent secondary antibody. Representative images from three independent experiments. Scale bar 10 μM, inset 4 μM. (C) FLAG-EP2 HEK 293 cells were incubated with/without dyngo-4a (50 µM; 45 min) treatment prior to live labelling with anti-FLAG M1 antibody before fixation and stained with immunofluorescent secondary antibody. Representative image is shown; *n* = 10 cells per condition. Scale bar 10 μM, inset 4 μM.

To explore potential mechanisms of ligand-independent EP2 internalisation, the requirement of β-arrestins and dynamin were assessed. Previously characterised HEK 293 cells where β-arrestin 1/2 are genetically ablated via CRISPR/Cas9 (Grundmann *et al.* 2018) were confirmed via western blotting (Fig. 2A and Supplementary Fig. 2). β-arrestin 1/2 knockout and wild-type HEK 293 cells were transfected with FLAG-EP2, and flow cytometry was performed to determine the cell surface levels of FLAG-EP2 in each cell type. Both the percentage of cells expressing the receptor and the mean intensity of each cell expressing the receptor were measured and found to be equivalent (Supplementary Fig. 3A and B). To assess internalisation, cells transiently expressing EP2 were assessed via confocal microscopy. Internalised receptor under basal conditions was still evident, suggesting that β-arrestin 1/2 is not involved in the constitutive internalisation of EP2 (Fig. 2B). However, pre-treatment of EP2-expressing cells with the dynamin inhibitor dyngo-4a inhibited basal EP2 internalisation, suggesting that dynamin is required for EP2 constitutive endocytosis (Fig. 2C).

#### Endosomal organisation of constitutively internalised EP2

**Figure 3:**
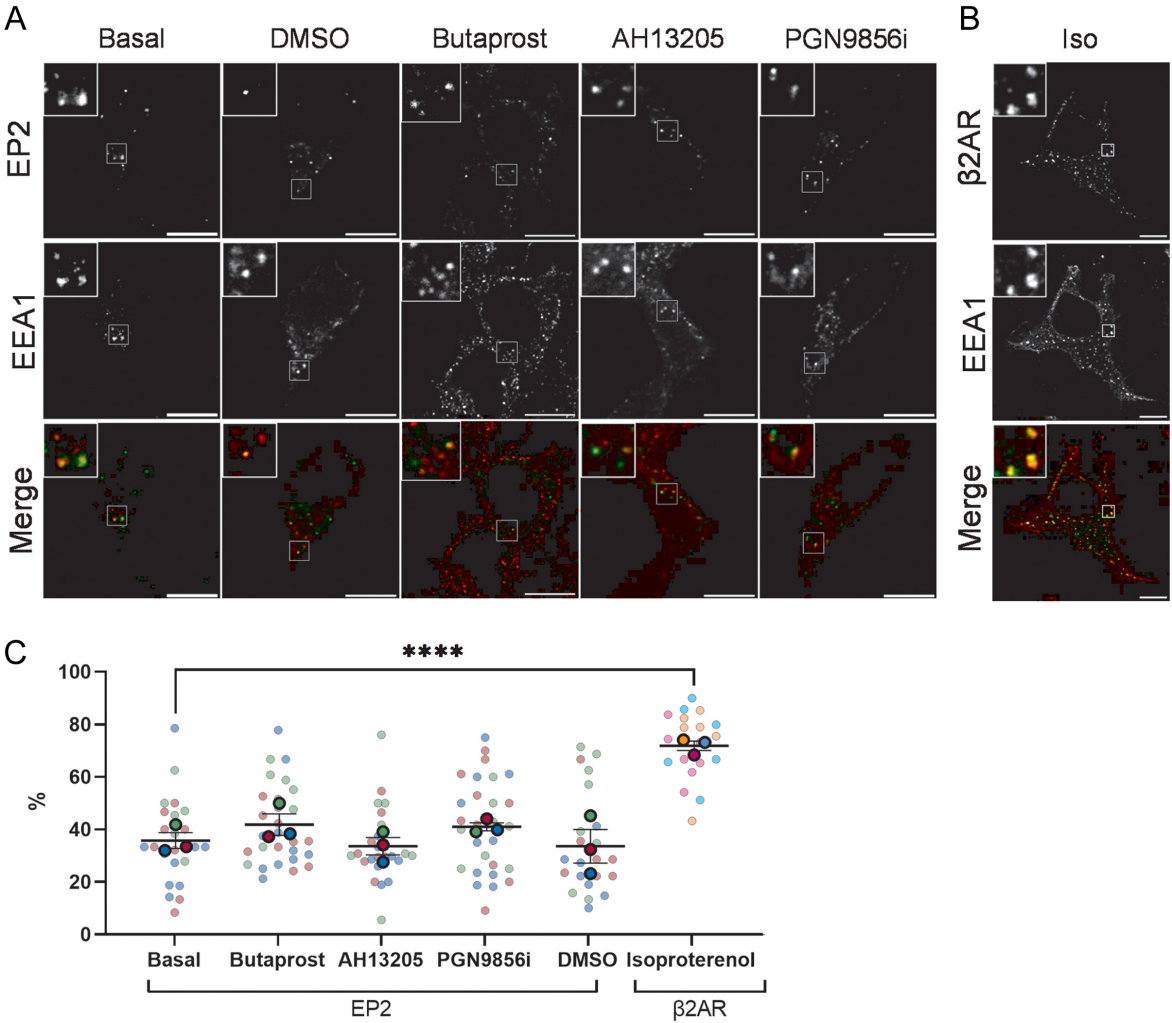
EP2 partially colocalises with early endosomes. (A,B) HEK 293 cells stably expressing (A) FLAG-EP2 or (B) FLAG-β2AR were live-labelled with anti-FLAG Ab and treated with either butaprost (10 μM), AH13205 (10 μM), PGN9856i (100 nM), DMSO or isoproterenol (10 μM) for 20 min. Cells were stripped of plasma membrane signal using EDTA, fixed, permeabilised and labelled with anti-APPL1 primary antibody and immunofluorescent secondary antibodies. Cells were imaged on a confocal microscope. Representative images shown with the receptor in the green channel and EEA1 in the red channel. Scale bar 10 μM. Inset size 4 μM. (C) Receptor-positive endosomes were counted using ImageJ, and the data are the percentage of receptor-positive endosomes that are colocalised with EEA1-positive endosomes. Data are mean ± s.e.m., *n* = 3, with the mean of each experiment shown as large circles and individual cell data shown as smaller circles, coloured for each experiment. One-way ANOVA with Dunnett’s multiple comparisons test: ***P < 0.001.

To identify the endosomal population that EP2 is sorted too, internalised receptor was visualised by confocal microscopy, and the degree of colocalisation between EP2 and a marker of the early endosome, early endosome antigen 1 (EEA1), was assessed (Fig. 3). Colocalisation of receptor and EEA1 was compared for EP2 across distinct ligand treatments to determine if ligand activation altered endosomal sorting. The endosomal organisation of EP2 was also compared against the β2AR, which following agonist treatment is known to robustly traffic to early endosomes (Fig. 3B). Quantitation of colocalisation demonstrated that ~36% of EP2 endosomes were positive for EEA1, and this was not significantly changed following activation with any of the ligands (Fig. 3C). Internalised β2AR primarily localised to an EEA1-positive compartment with ~68% of receptor trafficking to early endosomes following treatment with isoproterenol. This is in agreement with previous studies (Jean-Alphonse *et al.* 2014).

**Figure 4:**
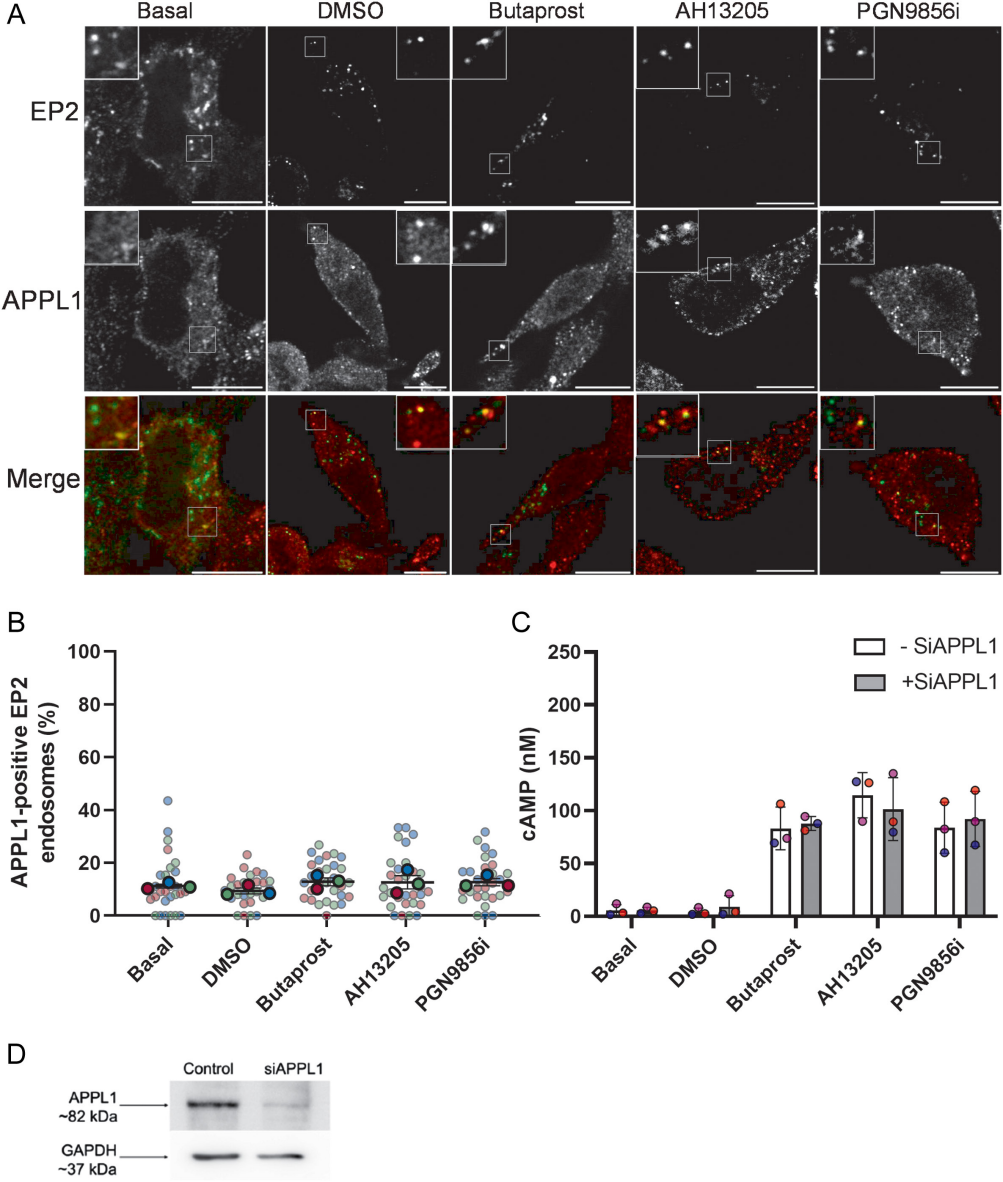
EP2 is localised to the very early endosome but is not regulated by APPL1. (A, B) HEK 293 cells stably expressing EP2 were live-labelled with anti-FLAG Ab and treated with butaprost (10 μM), AH13205 (10 μM), PGN9856i (100 nM) or DMSO for 20 min. Cells were stripped of plasma membrane signal using EDTA, fixed, permeabilised and labelled with anti-APPL1 primary antibody and immunofluorescent secondary antibodies. Cells were imaged on a confocal microscope. (A) Representative images shown with the receptor in the green channel and EEA1 in the red channel. Scale bar 10 μM. Inset size 4 μM. (B) EP2-positive endosomes were counted using ImageJ, and the data are the percentage of EP2-positive endosomes that are colocalised with APPL1-positive endosomes. Data are mean ± s.e.m., *n* = 3, with the mean of each experiment shown as large circles and individual cell data shown as smaller circles, coloured for each experiment. (C) APPL1 was knocked down using SiRNA and cAMP accumulation was measured after 5 min ligand treatment. (D) Representative western blot of APPL1 knockdown for Figure 4C.

Since the percentage of internalised EP2 that trafficked to early endosomes was significantly lower than β2AR, the presence of EP2 in a distinct endosomal compartment, the VEE, was explored. The VEE is physically smaller than early endosomes and lacks classic early endosomal markers such as Rab5 and EEA1. Furthermore, a subpopulation of these endosomes contains the adaptor protein, APPL1 (adaptor protein, phosphotyrosine interacting with PH domain and leucine zipper 1) that has been shown to have significant roles in both recycling of VEE-localised GPCR, and negative regulation of endosomal G protein signalling (Sposini *et al.* 2017, Caengprasath *et al.* 2020). The ability of internalised EP2 to colocalise with APPL1 was measured either with or without ligand activation. Under basal conditions, ~12% of EP2 endosomes colocalised with APPL1, and this was not altered with ligand activation (Fig. 4A and B). As APPL1 has been shown to negatively regulate Gαs-cAMP signalling for GPCRs that are regulated by the VEE (Sposini *et al.* 2017, Caengprasath *et al.* 2020), APPL1 was knocked out by siRNA and cAMP accumulation was measured (Supplementary Fig. 4 and Fig. 4C and D). There was no change in the ability of EP2 to induce cAMP production following activation with any ligand, suggesting that APPL1 does not play a role in EP2-cAMP signalling in HEK 293 cells.

#### EP2-mediated activation of Gαs and Gαq/11 signalling pathways are differentially regulated by internalisation

**Figure 5:**
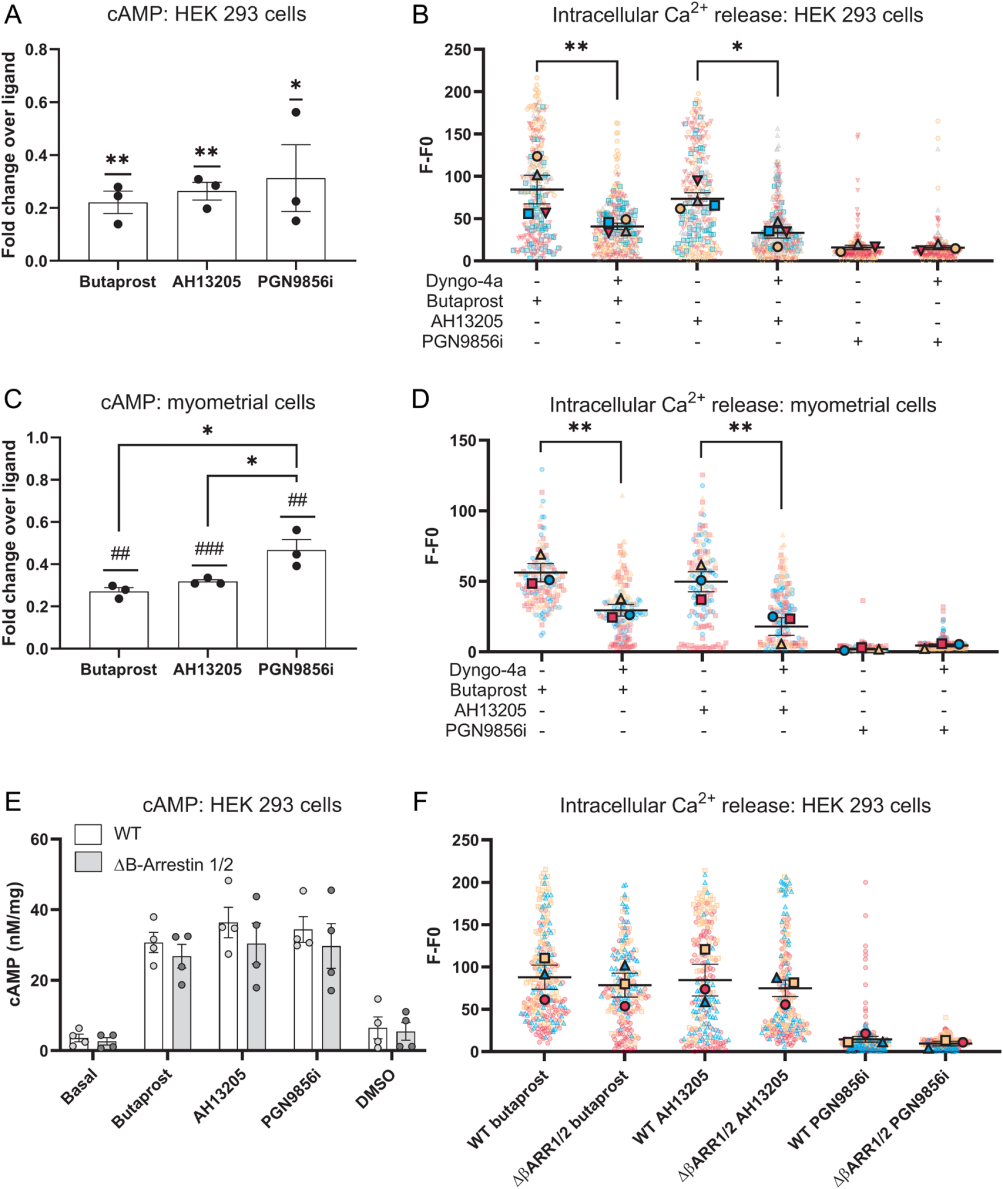
Internalisation via β-arrestin-independent dynamin-dependent pathways is required for full EP2 Gαs and Gαq/11 signalling. (A) HEK 293 cells expressing FLAG-EP2 incubated ± dyngo-4a (50 μM; 45 min) before an IBMX pretreatment (5 min, 500 μM) and 5 min stimulation with either butaprost (10 μM), AH13205 (10 μM) or PGN9856i (100 nM). Data shown normalised to control (ligand without dyngo-4a) and shown as mean ± s.e.m., *n* = 3. One-sample *t*-test: *P < 0.05. (B) HEK 293 cells expressing FLAG-EP2 were treated for 15 min with dyngo-4a (50 μM) then incubated with Fluo4 AM calcium indicator ± dyngo-4a for 30 min and intracellular calcium mobilisation was measured following acute stimulation with either butaprost (10 μM), AH13205 (10 μM) or PGN9856i (100 nM). Data shown as fluorescent intensity normalised to background for each cell measured (F–F0) and spots for each cell measured, colour coded to reflect inter-experimental variation. *n* = 3–4 independent experiments. One-way ANOVA with Šídák's multiple comparisons test: *P < 0.05, **P < 0.01. (C) cAMP accumulation was measured in primary myometrial cells as in Figure 5A. Data shown normalised to control (ligand without dyngo-4a) and shown as mean ± s.e.m., *n* = 3. One-way ANOVA with Tukey’s multiple comparisons test: *P < 0.05. One sample *t-*test: ^##^*P* < 0.01, ^###^*P* < 0.001. (D) Calcium mobilisation was measured in primary myometrial cells using the same protocol and analysis as Fig. 5B. *n* = 3 independent experiments, data shown as mean ± s.e.m. One-way ANOVA with Šídák's multiple comparisons test: *P < 0.05. (E) FLAG-EP2 expressing WT HEK 293 cells and cells lacking β-arrestin 1/2 were incubated with IBMX (5 min, 500 μM) before 5 min stimulation with either butaprost (10 μM), AH13205 (10 μM) or PGN9856i (100 nM). cAMP concentrations were normalised to protein and data shown as mean ± s.e.m., *n* = 3. (F) Calcium mobilisation was measured in primary myometrial cells using the same protocol and analysis as Fig. 5D. *n* = 3 independent experiments, data shown as mean ± s.e.m.

To assess the role of dynamin-dependent constitutive receptor internalisation on ligand-dependent EP2 signalling, acute ligand-mediated cAMP (Fig. 5A) and intracellular calcium release (Fig. 5B) was measured following dyngo-4a pre-treatment. Unexpectedly, the majority of the ligand-induced cAMP signal was lost following dyngo-4a treatment, suggesting a requirement for EP2 internalisation in acute cAMP signalling (Fig. 5A), as previously reported for LHR and FFA2 (Sposini *et al.* 2017, Caengprasath *et al.* 2020). Dynamin inhibition also significantly inhibited levels of intracellular calcium induced by butaprost and AH13205, whereby a ~50% reduction in ligand-induced increases in intracellular calcium was measured. PGN9856i ligand induced no increase in intracellular calcium (as previously reported by Walker *et al.* (2022)), either with or without dyngo-4a pretreatment (Fig. 5B and Supplementary Fig. 5).

To determine whether receptor signalling was spatially controlled in a physiological cell system, similar experiments were undertaken in term pregnant human myometrial cells as an established primary cell system that endogenously expresses EP2 and where EP2-selective ligands activate both cAMP and calcium signal pathways (Kandola *et al.* 2014, Walker *et al.* 2022). As was observed in HEK 293 cells, ~70% of ligand-induced cAMP required internalised receptors following treatment with either butaprost or AH13205 treatment; however, only ~54% of the cAMP signal was dependent on internalisation following activation with PGN9856i, highlighting potential differences in the compartmentalisation of signalling between the prostanoid agonists, butaprost and AH13205 and the non-prostanoid PGN9856i (Fig. 5C). Measuring intracellular calcium release following EP2 ligand stimulation also produced similar profiles as HEK 293 cells, whereby a ~50% reduction in calcium signalling occurred following dyngo-4a pretreatment (Fig. 5D).

Although β-arrestins were not required for EP2 internalisation, it is possible that they could play a role in signalling, as has been observed previously (Thomsen *et al.* 2016). cAMP accumulation (Fig. 5E) and calcium production (Fig. 5F and Supplementary Fig. 6) were measured in wild type and β-arrestin 1/2 knockout cells following ligand activation. No differences were observed between cell types in either ligand-induced intracellular cAMP or calcium levels, suggesting that β-arrestins may not regulate EP2-mediated Gαs or Gαq/11 signalling.

Overall, these results demonstrate a requirement for dynamin-dependent, arrestin-independent internalisation of the receptor for acute EP2 signalling and reveal potential distinct ligand dependencies for both Gαs-cAMP signaling and between G protein pathways.

#### EP2 agonist-mediated PGE2 secretion is internalisation independent

**Figure 6:**
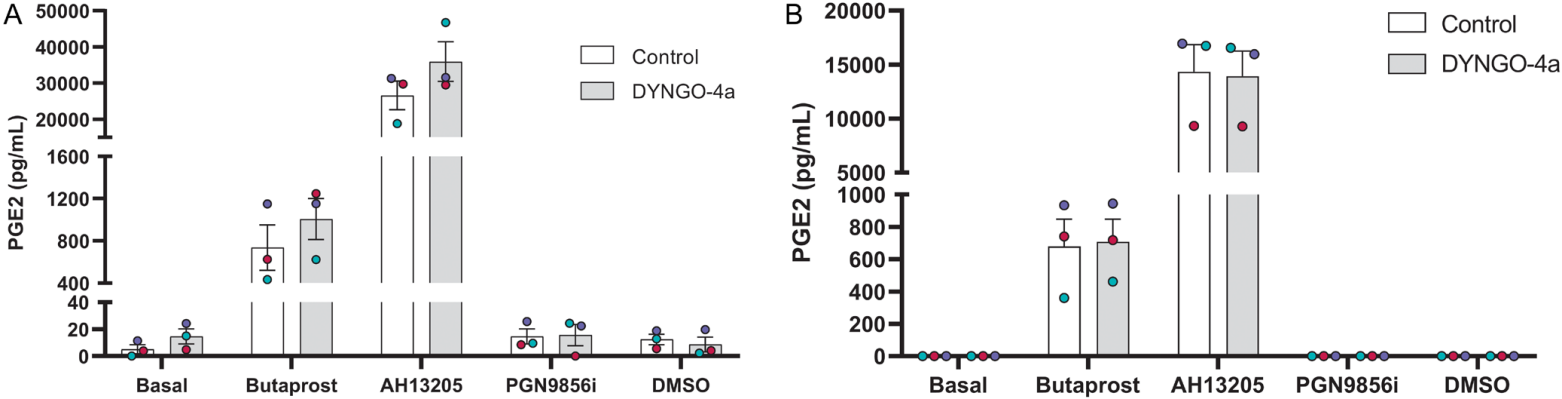
EP2-mediated PGE2 is released acutely and is not dependent on internalisation. HEK 293 cells stably expressing EP2 were pre-treated with/without dyngo-4a (45 min; 50 μM) before ligand stimulation with butaprost (10 μM), AH13205 (10 μM), PGN9856i (100 nM) or DMSO for either (A) 6 h or (B) 5 min. Media was removed after treatment and PGE2 concentration was determined by ELISA. Data are mean ± s.e.m., *n* = 3.

EP2-mediated PGE2 secretion is regulated by the Gαq/11–calcium–COX-2 pathway and differentially induced by distinct EP2 ligands (Kandola *et al.* 2014, Walker *et al.* 2022). As EP2-dependent calcium signalling partially required receptor internalisation (Fig. 5D), the spatial regulation of PGE2 secretion was measured. PGE2 secretion was measured in HEK 293 cells stably expressing EP2 following 6-h ligand stimulation, a timepoint we have previously employed to demonstrate EP2 agonist-mediated increases in COX-2 levels and PGE2 release in myometrial cells (Kandola *et al.* 2014, Walker *et al.* 2022), and following 5 min, to assess acute PGE2 secretion (Fig. 6B). At both timepoints, PGN9856i did not induce PGE2 secretion, consistent with our prior reports in term pregnant myocytes (Walker *et al.* 2022). Interestingly, AH13205 induced a significantly greater increase in PGE2 levels compared to butaprost. However, at both timepoints, there was no effect of dyngo-4a pre-treatment on either ligand-induced PGE2 secretion (Fig. 6B), suggesting that EP2-mediated PGE2 production and secretion do not require receptor internalisation.

## Discussion

EP2 is critically involved in a broad range of physiological and pathophysiological functions. The identification that certain EP2 ligands, including PGE2, can induce not only Gαs-cAMP signalling but also Gαq-Ca^2+^ signalling may underlie its pleiotropic actions in distinct tissues. However, the regulation of these two pathways is poorly understood. In this study, we examined the role of receptor internalisation on these pathways and identified that EP2 constitutively internalises to distinct endosomal populations, and that although trafficking is not altered by ligand treatment, this constitutive internalisation is required for driving EP2 acute G protein signalling.

The present study confirms previous reports that EP2 does not undergo agonist-driven internalisation (Desai *et al.* 2000, Penn *et al.* 2001) but identifies constitutive internalisation of EP2. This is supported by previous studies which find that generally, there is an inverse relationship between constitutive and agonist-induced internalisation (Hendrik Schmidt *et al.* 2020). This has been overlooked for EP2, as studies have focused exclusively on agonist-driven internalisation (Desai *et al.* 2000, Penn *et al.* 2001). EP2 internalisation occurred in a β-arrestin-independent, but dynamin-dependent manner, which contrasts with EP4, another Gαs-coupled EP receptor, which robustly internalises and signals via β-arrestin (Penn *et al.* 2001, Buchanan *et al.* 2006), yet is consistent with previous studies demonstrating that agonist-activated EP2 is not regulated by arrestins due to the lack of C-terminal tail phosphorylation sites (Penn *et al.* 2001, Choi Kong *et al.* 2008). This suggests that inability to undergo phosphorylation by GRKs prevents EP2–β-arrestin interaction. The receptor-dependent sequences that drive its constitutive internalisation may provide molecular insight into the β-arrestin-independent yet dynamin-dependent properties of this internalisation. Whilst C-terminal tail sequences may be sufficient to modify the internalisation of other GPCRs employing ligand and β-arrestin-dependent mechanisms (Wanka *et al.* 2017), a more systematic approach may be required for EP2 to consider additional structural and post-translational modifications that drive its constitutive and β-arrestin-independent trafficking.

EP2 was found to partially traffic to early endosomes and, to a lesser extent, the APPL1 component of the VEE compartment, which is consistent with APPL1 exhibiting no negative regulation of EP2-mediated Gαs-cAMP signalling. However, given the small size of EP2 endosomes, low levels of co-localisation with EE markers, and that very little is known about additional protein machinery within the VEE, it is possible that distinct endosomal proteins regulate internalisation-dependent signalling of EP2.

However, despite the ability of distinct EP2 ligands such as PGN9856i to induce different G protein signalling compared to butaprost and AH13205, they did not induce distinct trafficking profiles of EP2, and all EP2 ligand-induced signalling was both β-arrestin- and APPL1-independent. Given that internalisation occurs in the absence of ligand activation, this may suggest that conformations induced by these agonists to induce G protein activation are uncoupled from conformations that induce receptor internalisation. Indeed, while EP2 exhibits basal internalisation, no constitutive signalling was evident. Therefore, EP2 could have a conserved trafficking profile that functions to modulate and maintain EP2 signalling and expression, regardless of ligand activation. This is supported by data showing that EP2 is not desensitised during prolonged stimulation, suggesting that the receptor is recycled back to the surface membrane, enabling EP2 to be activated over prolonged periods (Desai *et al.* 2000, Nunez *et al.* 2020). There is a precedent for this mechanism in other receptors, such as GPRC6A, a class C GPCR which undergoes constitutive internalisation that is not altered by agonist treatment and traffics via the Rab11-dependent slow recycling pathway to maintain plasma membrane expression (Jacobsen *et al.* 2017).

Previous work has shown that GPCRs with Gαq/11 activity are more likely to constitutively internalise (Hendrik Schmidt *et al.* 2020). Both this and previous studies from our group and others have shown crucial roles for EP2-mediated Gαq/11-calcium signalling, despite this receptor classically signalling via Gαs (Kandola *et al.* 2014, Kishore *et al.* 2017, Walker *et al.* 2022). Functional studies employing a dynamin inhibitor to prevent internalisation revealed that activation of both the Gαs and Gαq/11 pathways requires internalised receptor, although to different extents. The role of endosomal Gαq signalling in the absence of relevant lipid species in the early endosomal membrane to generate the necessary second messengers from PLC activation is a topic of current discussion, even though Gαq is present and activated at the endosome (Wright *et al.* 2021). The VEE endomembrane, while yet to be characterised completely, is not dominated by PI3P characteristic of the early endosomal membrane (Jean-Alphonse *et al.* 2014), and thus may facilitate endosomal Gαq/PLC signalling that utilises PIP, or even alternate lipids may activate PLC, such as PI4P known to be present in Golgi and recycling endosomes (de Rubio *et al.* 2018).

Whilst this study identified receptor internalisation as a critical component of EP2 signalling, the intracellular location of EP2-Gαs or Gαq signalling remains to be determined. Ligand-dependent EP2 elevation of cAMP is a critical pathway for this receptor, a pathway, and indeed second messenger, that mediates diverse functions depending on many factors, including the cellular/tissue environment. One key, well-described function of EP2-mediated cAMP signalling is smooth muscle relaxation. Indeed, butaprost and PGN9856 inhibit the contractility of human uterine smooth muscle strips (Coleman *et al.* 2019). It is possible that distinct functions, such as contractility, require mechanisms of spatial control to differentiate cAMP signals in the same cell type to mediate distinct functions.

This study may suggest different roles of EP2-mediated plasma membrane and intracellular Gαq/11-calcium signalling as PGE2 secretion, previously shown to require EP2-dependent calcium signalling (Kandola *et al.* 2014), did not require EP2 internalisation, suggesting the signals that drive PGE2 secretion likely emanate from the plasma membrane. Interestingly, AH13205 induced significantly higher PGE2 secretion than butaprost (36-fold more), and could be expected to elicit an enhanced, more efficacious calcium signalling response compared to butaprost, which was not the case. This suggests additional mechanisms downstream of calcium signalling are involved. Both acute and chronic PGE2 release was detected in response to EP2 ligand treatment (butaprost and AH13205), and COX-1 and COX-2 are expressed at very low levels in HEK293 cells with prostaglandins reportedly not stored in this cell type (Funk 2001, Ricciotti & FitzGerald 2011), one possibility is a role of EP2 agonist-dependent modulation, and in particular AH13205, of phospholipase A2 activity, the rate-limiting step in the PGE2 production pathway.

Overall, EP2 exhibits constitutive internalisation to distinct endosomal compartments, and this internalisation is required for normal acute G protein signalling by distinct EP2 ligands. The potential differential spatial requirements of EP2-mediated cAMP and calcium signalling, and how a cell may decode this signalling at a spatial level, remains to be determined. However, these findings suggest that efficacy of EP2 agonists to selectively EP2-mediated physiological, or pathophysiological functions could be enhanced by directing the location of EP2 activity towards plasma membrane or endosomal signalling. This, in turn, may inform the basis for novel pathway selective therapeutic strategies.

### Limitations

Primary myometrial cells were used as a previously published model of endogenous EP2 signalling (Kandola *et al.* 2014, Walker *et al.* 2022). Although no differences were observed in the spatial control or signal profiles of EP2 between HEK 293 cells and myometrial cells, it is possible that progesterone and/or other hormones that the myometrium is exposed to could result in differences in regulation of trafficking and the spatial control of EP2. We currently do not understand how each EP2 ligand employed in this study may induce distinct conformational states to not only activate distinct signal profiles but also induce differential regulation at a spatial level. Whilst there is one available high-resolution structure of PGE2-bound EP2, future studies will require additional structural information that incorporates both space and time into our understanding of the molecular basis of ligand-directed signalling of GPCRs.

## Supplementary Materials

Supplementary Figures

## Declaration of interest

An international patent application for the method of use of PGN9856i in preterm labour described in this article has been filed (PCT/GB2021/051971) on behalf of ARW, PRB and ACH by Imperial College London. The remaining authors declare no competing interests.

## Author contribution statement

ACH conceived the study and with ARW designed experiments. HAP performed experiments and analysed datasets for Figure 4C–D, under the supervision of ARW and ACH. ARW performed all other experiments and analysed all data sets under the supervision of ACH and PRB. DFW provided a series of compounds from which PGN9856i was selected, by consensus, for study. VT consented patients and with SK provided all non-labouring myometrial cultures. ARW and ACH wrote the manuscript and all authors contributed and approved the final version.

## Funding

We would like to acknowledge the following funding bodies for making this work possible: Society for Endocrinologyhttp://dx.doi.org/10.13039/501100000382 Early Career Grant (ARW); Genesis Research Trusthttp://dx.doi.org/10.13039/100012156 (grant number P65251) (ACH); the National Institute for Health Researchhttp://dx.doi.org/10.13039/100005622 Biomedical Research Centre (NIHR BRC) based at Imperial Healthcare NHS Trust (grant no. P45272) (PRB).
