# Supplementary material for: Constitutive internalisation of EP2 differentially regulates G protein signalling
Source: J Mol Endocrinol. 2024 May 15;73(1):e230153. doi: 10.1530/JME-23-0153 (PMC11227035; doi:10.1530/JME-23-0153)
Supplement: Supplementary Figures [file supplementary_figures.pdf]

Supplemental figures for: Constitutive internalisation of EP2 differentially regulates G protein signalling

Authors: Abigail R. Walker[1]*, Holly A Parkin[1], Sung Hye Kim[1], Vasso Terzidou[1], David F. Woodward[2], Phillip R. Bennett[1], Aylin C. Hanyaloglu[1]*

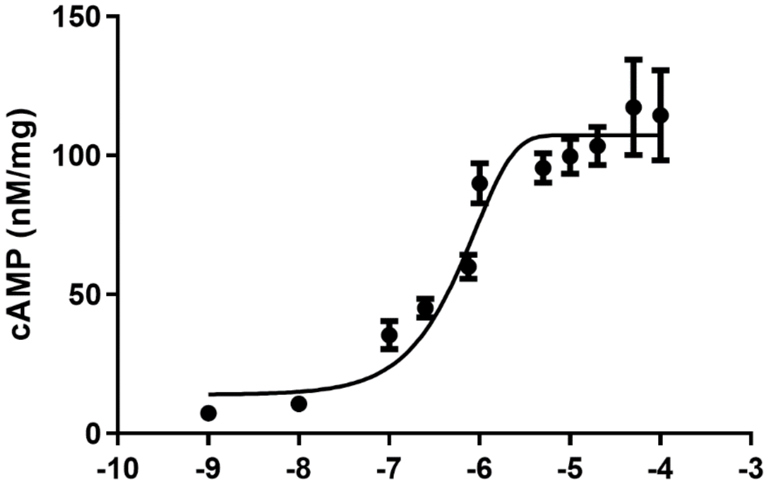

Supplemental Figure S1. Dose-response profile for AH1205-induced cAMP in HEK 293 cells expressing EP2. HEK 293 cells stably expressing EP2 were treated with IBMX (5 min, 0.5mM) before stimulation with IBMX and AH13205 for a further 5 min. cAMP concentrations were normalised to protein. Data represent mean ± SEM from 3 independent experiments.

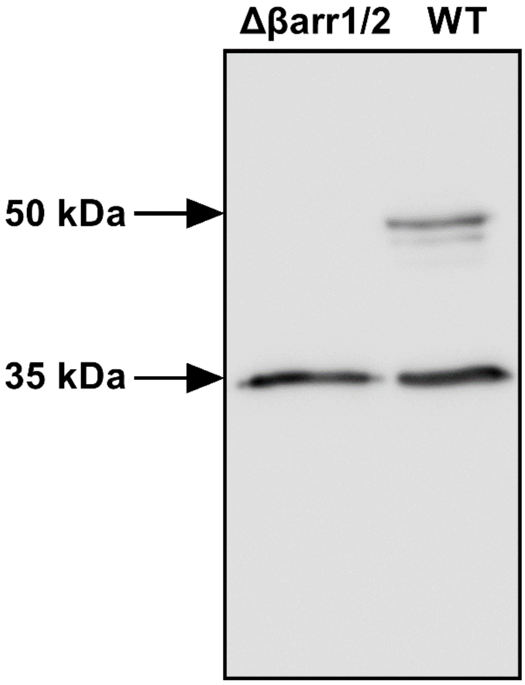

Supplemental Figure S2. β-arrestin1/2 is not detectable in β-arrestin 1/2 knockout cells. Uncropped western blot related to Figure 2A. Lysates of WT HEK 293 cells and cells lacking β-arrestin 1/2 were assessed via western blot for cellular levels of β-arrestin 1/2 protein (~50 kDa) using anti-GAPDH as a loading control (~35 kDa). Representative image shown.

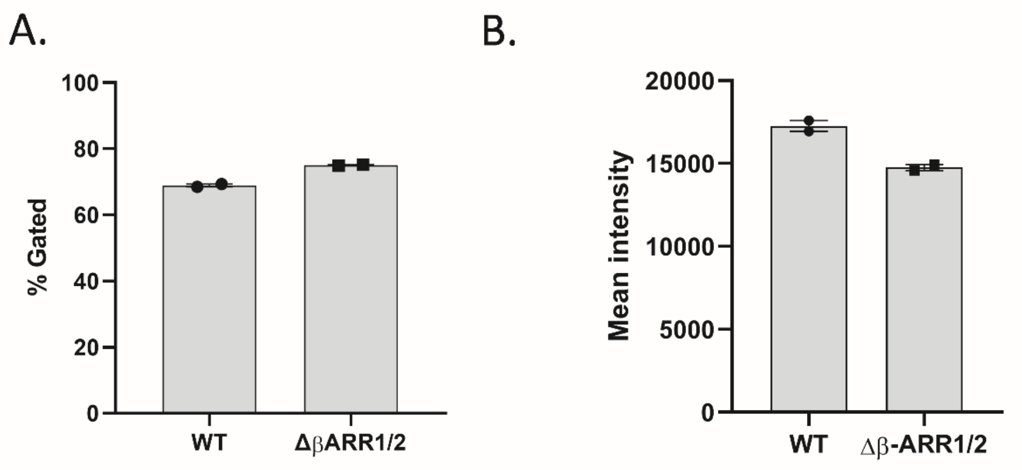

Supplemental Figure S3. Wild-type HEK 293 and β-arrestin 1/2 knockout cells express similar levels of cell surface FLAG-tagged EP2. A-B) WT HEK 293 and cells lacking β-arrestin 1/2 were transiently transfected with FLAG-EP2 and cell surface levels of receptor assessed via anti-FLAG staining and flow cytometry, n=1 with two technical replicates. **(A)** The percentage of cells expressing receptor **(B)** and the mean fluorescence intensity of cells expressing EP2**.** Related to Figure 2A-B.

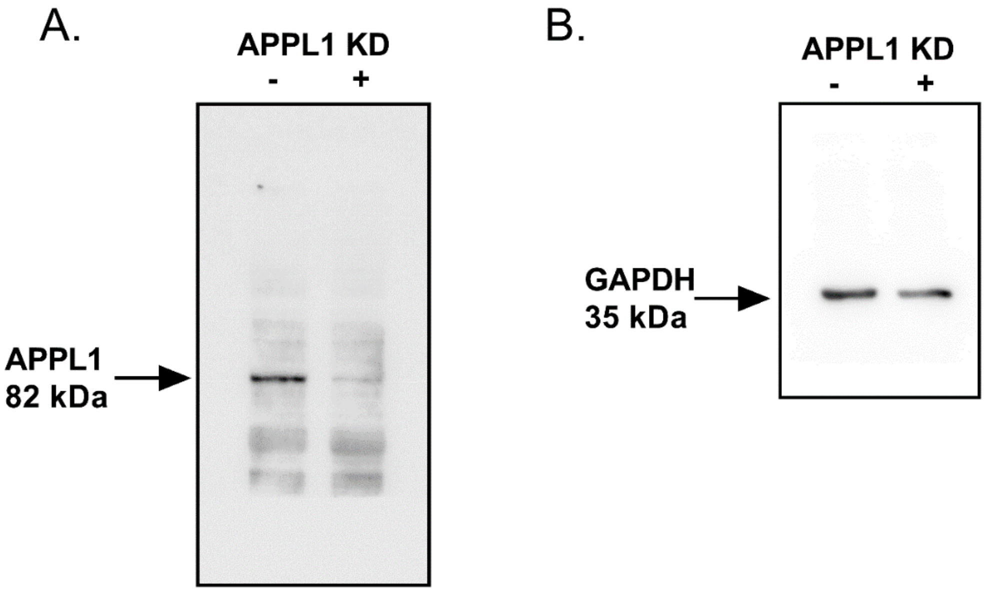

Supplemental Figure S4. APPL1 SiRNA knockdown in HEK 293 cells. Uncropped representative western blots relating to Figure 4. Cellular levels of APPL1 following treatment with or without APPL1 siRNA were measured via Western blot. Membrane was cut to incubate with either (**A)** anti-APPL1 antibody (~82 kDa) or **(B)** anti-GAPDH antibody (~35 kDa), as a loading control. Representative images shown.

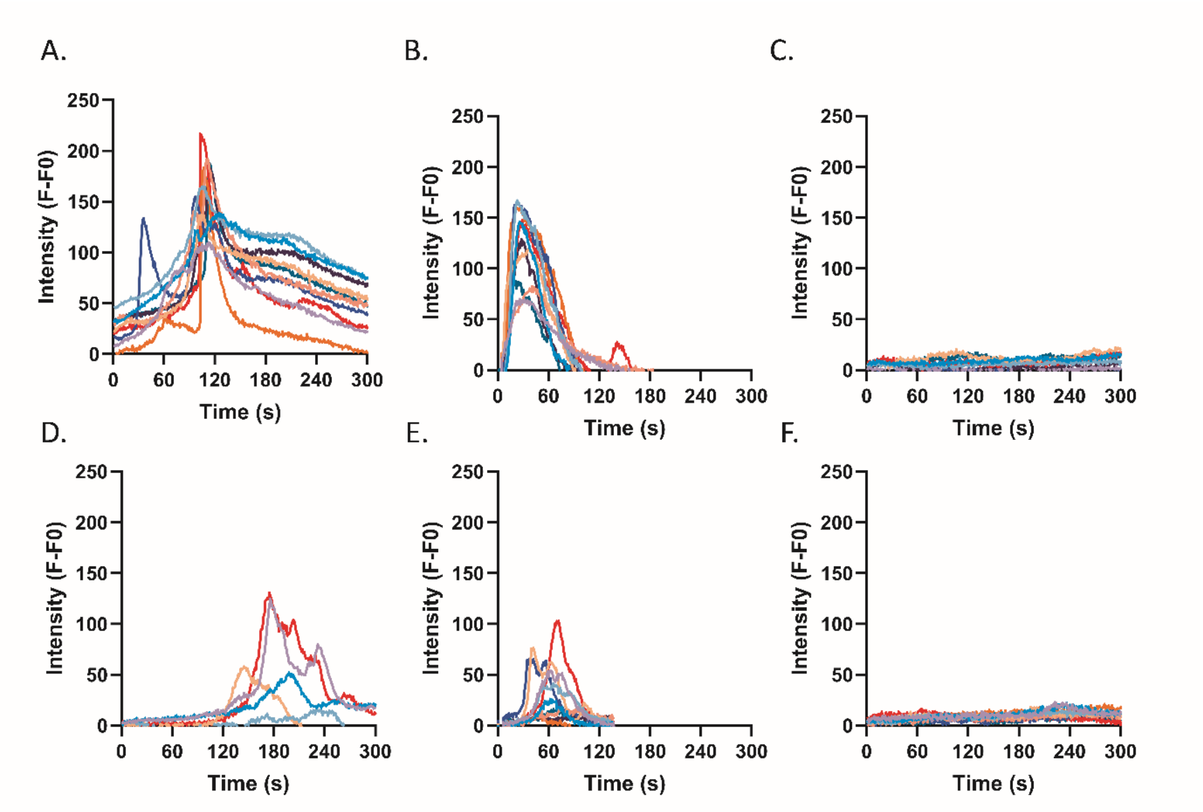

Supplemental Figure S5. Intracellular calcium release is dynamin dependent. HEK 293 cells expressing FLAG-EP2 were treated for 15 min with dyngo-4a (50 μM) then incubated with Fluo4 AM calcium indicator ± dyngo-4a for 30 min and intracellular calcium mobilisation was measured following acute stimulation with either butaprost (10 μM), AH13205 (10 μM) or PGN9856i (100 nM). Data is the profile of fluorescent intensity normalised to background for 10 representative cells (F-F0). Related to Figure 5B. **A)** Butaprost (10 μM) **B)** AH13205 (10 μM) **C)** PGN9856i (100 nM) **D)** Butaprost (10 μM) + dyngo-4a **E)** AH13205 (10 μM) + dyngo-4a **F)** PGN9856i (100 nM) + dyngo-4a.

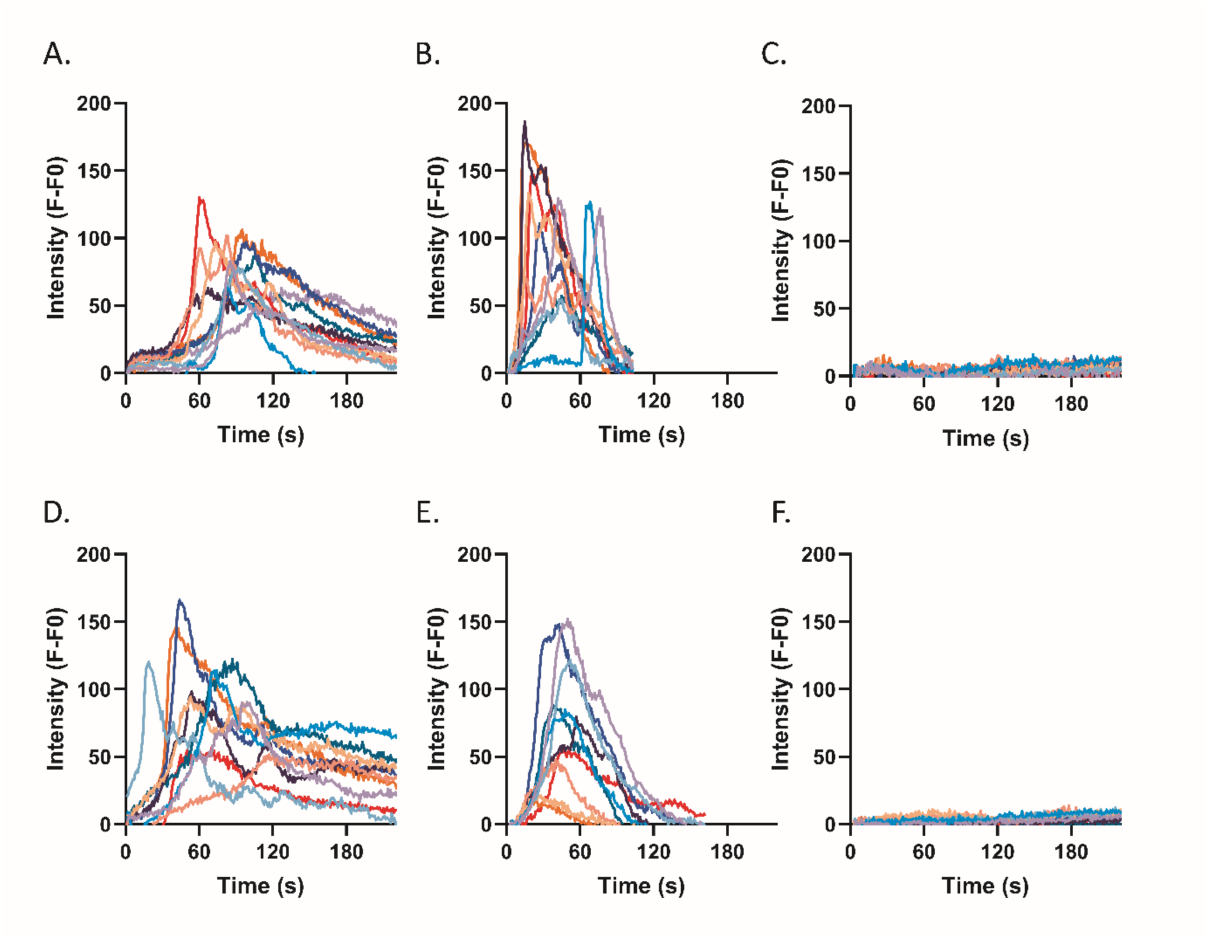

Supplemental Figure S6. β-arrestin 1/2 knockout does not alter the temporal profile of intracellular calcium release. HEK 293 cells and β-arrestin 1/2 knockout cells were transiently transfected with FLAG-EP2 and incubated with Fluo4 AM calcium indicator for 30 min and intracellular calcium mobilisation was measured following acute stimulation with EP2 ligands. Data is the profile of fluorescent intensity normalised to background for 10 representative cells (F-F0). Related to Figure 5F. **A-C)** HEK 293 cells activated with **A)** Butaprost (10 µM) **B)** AH13205 (10 µM) **C)** PGN9856i (100 nM). **D-F)** β-arrestin 1/2 knockout cells activated with **D)** Butaprost (10 µM) E**)** AH13205 (10 µM) **F)** PGN9856i (100 nM).
